# Micronutritional status after pylorus preserving duodenopancreatectomy: analysis of data from a randomized controlled trial

**DOI:** 10.1038/s41598-021-97438-6

**Published:** 2021-09-16

**Authors:** Navid Tabriz, Verena Nicole Uslar, Dennis Obonyo, Dirk Weyhe

**Affiliations:** University Hospital for Visceral Surgery, Pius-Hospital Oldenburg, Carl Von Ossietzky University Oldenburg, Georgstr. 12, 26121 Oldenburg, Germany

**Keywords:** Randomized controlled trials, Pancreatic cancer, Surgical oncology

## Abstract

Physical frailty and nutritional malassimilation are often observed after pancreaticoduodenectomy for pancreatic cancer. But long-term data concerning the course of micronutrient status is still missing. Micronutrient status after pylorus preserving pancreaticoduodenectomy with a follow-up of 12 months was evaluated using data of a randomized controlled trial. 47 patients were randomized with respect to the physiotherapy regimen they received (intensified physiotherapy: n = 22; standard physiotherapy: n = 25). Nutritional status was recorded preoperatively and postoperatively after one week, 3, 6 and 12 months. BMI, body fat measurement and albumin, lipid, iron and bone metabolism parameters, vitamins A, B1 B6 and B12, homocysteine, folic acid, and trace elements were measured. Laboratory values were analyzed descriptively. Differences between the groups were analyzed using the t-test in SPSS. For vitamin D, B1, B6 and iron a deficiency over time could be demonstrated with 50% of all patients or more being below normal range. The other laboratory values were in low normal range after 3 months and later. Significant differences between groups were found in cholesterol, HDL and selenium levels (corrected *p*-values < 0.033 in all cases). Vitamin D and iron should be supplemented postoperatively in the long term, and vitamin B1 and B6 substitution should be considered in symptomatic patients. Levels of malnutrition induced fatigue should be comparable between both groups. However, the role of nutritional status on other health-related aspects such as quality of life should be the focus of further studies.

Trial Registration Number in the German Registry for Clinical Studies: DRKS00006786; Date of Registration: 01.10.2014.

## Introduction

According to the International Agency for Research on Cancer (IARC), pancreatic cancer is the 12th most common cancer worldwide and the fourth leading cause of cancer-related death^1–3^. Currently surgery combined with chemotherapy offers the only chance for cure, though with a dismal 5-year survival ranging from 8 to 17%^4^. Therefore, it seems of utmost importance to not only concentrate on overall survival rate of those patients, but to also address health-related issues like physical wellbeing to ensure good Quality of Life (QoL) for the remainder of their time. Physical wellbeing, in turn, depends on many different factors, including physical exercise and good nutrition. However, the absorption of micronutrients may be disturbed due to the classic Whipple´s or the pylorus preserving pancreaticoduodenectomy, which are currently the standard surgical procedures^5,6^. On the one hand, the surgical resection of the pancreas can lead to endo- and exocrine pancreas insufficiency. On the other hand, deficiencies of micronutrients (vitamins, minerals, trace elements) can occur due to the resection of the duodenum and proximal jejunum^7^. In addition, inappropriately treated pancreatic exocrine insufficiency can increase deficiencies of micronutrients, sarcopenia and even mortality^8^.

Therefore, it has been postulated that patients after pancreaticoduodenectomy (PD) might show deficiencies in several micronutrients including fat-soluble vitamins D and E, selenium and iron, independent of dietary intake or of pancreatic exocrine function, and therefore clinical recommendations for micronutrient evaluation have been issued^9,10^. But long-term data concerning the time course of micronutrient levels after cancer-related pancreatic head resection are missing^11^.

A routine examination of micronutrients of PD patients pre- and postoperatively is viewed critically as malignancy or infectious diseases can make serum estimation of micronutrients unreliable and therefore unrepeatable especially in the initial postoperative course^12^. Therefore, an evaluation of the micronutritional status should be conducted over a longer period to avoid potential influences of the operation itself and early postoperative inflammatory changes. In this study we used the data gathered throughout a randomized controlled trial performed in our clinic which investigated the influence of intensive physiotherapy as compared to standard physiotherapy on QoL as a primary outcome, and the course of the micronutritional status over a long follow-up period as a secondary outcome (see study protocol published by Richter et al.^13^ and the German Clinical Trials Registry (DRKS); Study ID: DRKS00006786).

The main aim of the analysis reported here was to evaluate how PD surgery influences nutritional status in a typical PD patient collective during a follow-up period of 12 months when no nutritional intervention is performed. To our knowledge, this is the first prospective study with such a long follow-up period conducted exclusively in pancreas cancer patients.

## Methods

### Study design

The data analyzed for this study was gathered during a prospective randomized controlled study including all consecutive patients with resectable pancreatic cancer, distal bile duct carcinoma, neuroendocrine tumors, periampullary carcinoma, duodenal carcinoma, and intraductal papillary mucinous neoplasms treated in the University Clinic for Visceral Surgery, Pius Hospital Oldenburg, University of Oldenburg during the study period (02/2016–11/2019). The primary aim of this RCT was to analyze the effect of intensive physiotherapy on QoL and the secondary aim was the evaluation of the micronutritional status over time. Here, we report the results of the micronutritional evaluation.

Informed written consent was obtained from all patients after the study had been explained to them. The study is registered with the German Clincal Trials Registry (DRKS; Study ID: DRKS00006786; Date of registration: 01/10/2014), it was approved by the ethics committee of the Carl von Ossietzky University Oldenburg (vote number: 59/2014), and it was performed in accordance with relevant guidelines and regulations, especially the CONSORT statement. For further details we also refer to our publication of the study protocol^13^. In summary, patients with suspected pancreatic head carcinoma were preoperatively randomized in an interventional group (intensified physiotherapy (IG)) and a classic group (standard physiotherapy(CG)). For this purpose, a MATLAB-based script was used that generated a list for 84 study enrollments with 6-block randomization with 1:1 allocation. Based on this list, 84 numbered envelopes were prepared by a person not directly involved in this study, each containing a slip of paper with the group assignment. These envelopes were opened in consecutive order in the presence of the patients after inclusion. In case of intraoperative inoperability due to local vascular invasion or carcinosis or postoperative benign histological results, patients were excluded from the study. Furthermore, only patients treated by pylorus preserving pancreaticoduodenectomy as described by Traverso/Longmire^14^ were included in the analysis.

Nutritional and metabolic status measurements were performed 2 days preoperatively and postoperatively after one week, 3 months, 6 months and 12 months. The measured parameters included weight, size, body mass index (BMI), body fat measurement by using the seven-site skinfold method and the following laboratory values: albumin, iron, transferrin, ferritin, vitamin B12, homocysteine, folic acid, magnesium, zinc, calcium, copper, selenium, parathyroid hormone, vitamin D, hemoglobin, vitamin A, cholesterol, high-density lipoprotein (HDL) cholesterol, low-density lipoprotein (LDL) cholesterol, and triglycerides. All laboratory values were measured based on blood plasma or serum samples as appropriate (see Supplemental Table 1 for a detailed overview of the test kits used).

Postoperative oral food intake was started as soon as it could be tolerated, and nutritional consultation was established during inpatient hospital stay. Patients were monitored for endocrine and exocrine insufficiency postoperatively. Food related lipase substitution (2,000 U Kreon/gram fat) was established during hospital stay and was checked and adjusted if necessary by patient´s general practitioner after discharge. Therefore, it could be ensured that the patients were well supplemented with pancreatic enzymes, and clinically relevant exocrine pancreatic insufficiency could be excluded for all patients. In the medium and long term, a balanced diet with a daily caloric intake of 25–30 kcal/kg body weight according to the ESPEN guidelines was recommended^15^, and the monthly phone calls and visits included general questions with regards to appetite and food intake, as well as the respective questions in the QoL questionnaires used in the study, namely the SF-8, the EORTC QLQ-C30, and the QLQ-PAN26 questionnaires in the validated German translation^16–19^. Patients included in the latter analysis all reported acceptable to normal food intake. None of the patients took additional nutritional supplements.

### Physiotherapy

The intensified physiotherapy started within the first 24 h after extubation with 3 rounds of bed cycling per day (10 min each). From the second postoperative week patients were supposed to walk 3 times/day (15 min each), and to do muscle exercises using a Theraband (resistance band), 2 kg dumbbells and modified squats 5 days per week. After discharge from the rehabilitation clinic to the 12th postoperative month, patients were encouraged to walk according to Every Step Counts graduated walking program with Yeo et al.´s minor modifications for resected pancreas cancer patients^20^, and they were asked to continue their muscle exercises at least 3 times/week.

The standard physiotherapy depended on the physical condition of the patient and was limited to the duration of the hospital stay and was scheduled for 20 min/day on 5 days per week. It consisted of individual therapy with relaxation and mobilization exercises, walking, and even climbing stairs. To minimize rehabilitation therapy as a confounding factor, all included patients received rehabilitation in the same clinic.

### Statistical analyses

Sample size calculation was performed for the primary endpoint of the RCT. Therefore, all parameters reported here were analyzed only descriptively with intention to treat analyses by calculating median and mean values as well as the 5% and 95% percentile and the 25% and 75% quartiles. Those values were depicted in boxplots (prepared with the graphing software Origin 2021 by OriginLab) and compared to the normal range of the respective parameter over the course of the follow-up. Values were judged to be within normal range if the box was completely within the area for the normal range, indicating that at least 50% of all gathered data was within normal range. Values were considered below or above the norm if the median was not within the normal range, indicating that less than 50% of the data were within the normal range. Values were considered above or below average if the entire box was outside the normal range, indicating that at least 75% of the data was not within the normal range.

To evaluate micronutritional status with respect to intervention and control group, t-tests for detection of differences between IG and CG patients were conducted using IBM SPSS Statistics software (Version 26). P-values- below 0.05 were regarded as statistically significant. Effect sizes are calculated to judge for sufficient sample size. Benjamini–Hochberg correction of the p-values was applied to adjust for multiple testing.

### Ethics approval and consent to participate

The study was approved by the ethics committee of the Carl von Ossietzky University Oldenburg (vote number: 59/2014). Informed written consent was obtained from all patients after the study had been explained to them. The ability to give written consent was a requirement for inclusion in this study.

## Results

**Figure 1:**
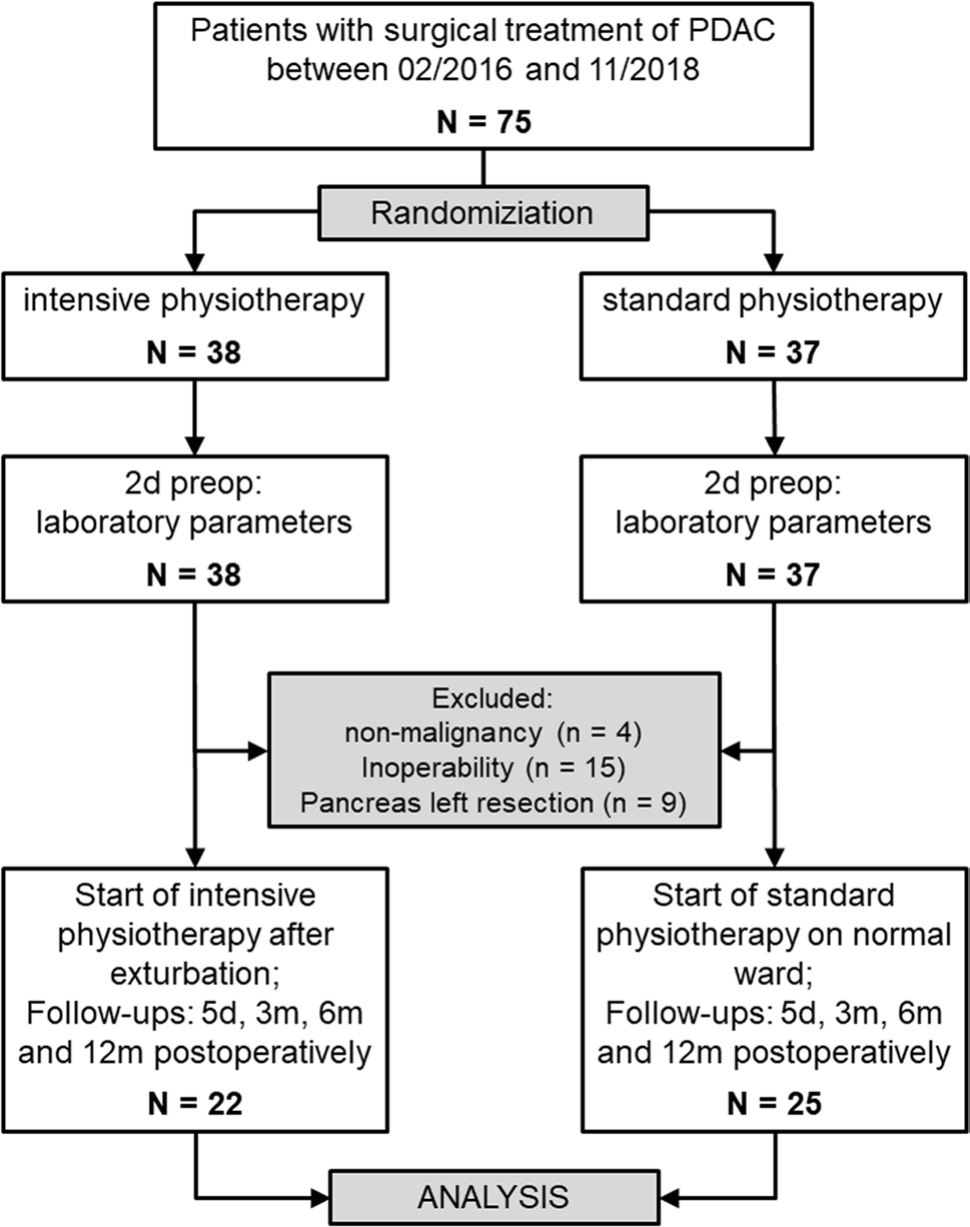
Study flow chart.

A total of 75 Patients were included in the study and randomized preoperatively to the interventional group (IG: n = 38) and the control group (CG: n = 37; see Fig. 1). After surgery, 19 patients were excluded because of non-malignancy or inoperability. Out of the remaining 56 patients, 9 patients received pancreas left resection, and were therefore excluded. Thus, n = 47 patients (n = 25 in CG and n = 22 in IG) were analyzed. Most patients (n = 40) were diagnosed with adenocarcinoma and 72% were classified in UICC stage II and III (see Table 1). In the 3 patients with stage IV (n = 2 NET and n = 1 adenocarcinoma), a singular liver metastasis was detected intraoperatively. After complete excision of the liver metastasis, the patients underwent pancreatic resection as planned. N = 31 (66%) of patients (n = 13/59.1% in IG and n = 18/72% in CG) received chemotherapy.

**Table 1:** Patient characteristics.

	Overall	Intensive physiotherapy	Standard physiotherapy
	n = 47	n = 22	n = 25
Sex (m/f)	26/21	14/8	12/13
Age (years; mean ± SD)	67.1 ± 10.1	69.1 ± 8.5	65.3 ± 11.2
BMI (mean ± SD)	26.7 ± 5.2	26.4 ± 4.5	27.0 ± 5.8
**Carcinoma type (n/%)**			
Adeno carcinoma	40/85.1	18/81.8	22/88.0
NET	4/8.5	3/13.6	1/4.0
IPMN	2/4.3	2/9.1	0/0
Acinar cell carcinoma	1/2.1	0/0	1/4.0
**UICC stage (n/%)**			
IA	2/4.3	1/4.5	1/4.0
IB	7/14.9	3/13.6	4/16.0
IIA	7/14.9	5/22.7	2/8.0
IIB	18/38.3	6/27.3	12/48.0
III	10/21.3	4/18.2	6/24.0
IV	3/6.4	3/13.6	0/0
Chemotherapy received (n/%)	31/66.0	13/59.1	18/72.0

### BMI (body mass index), body fat and lipid parameters

**Figure 2:**
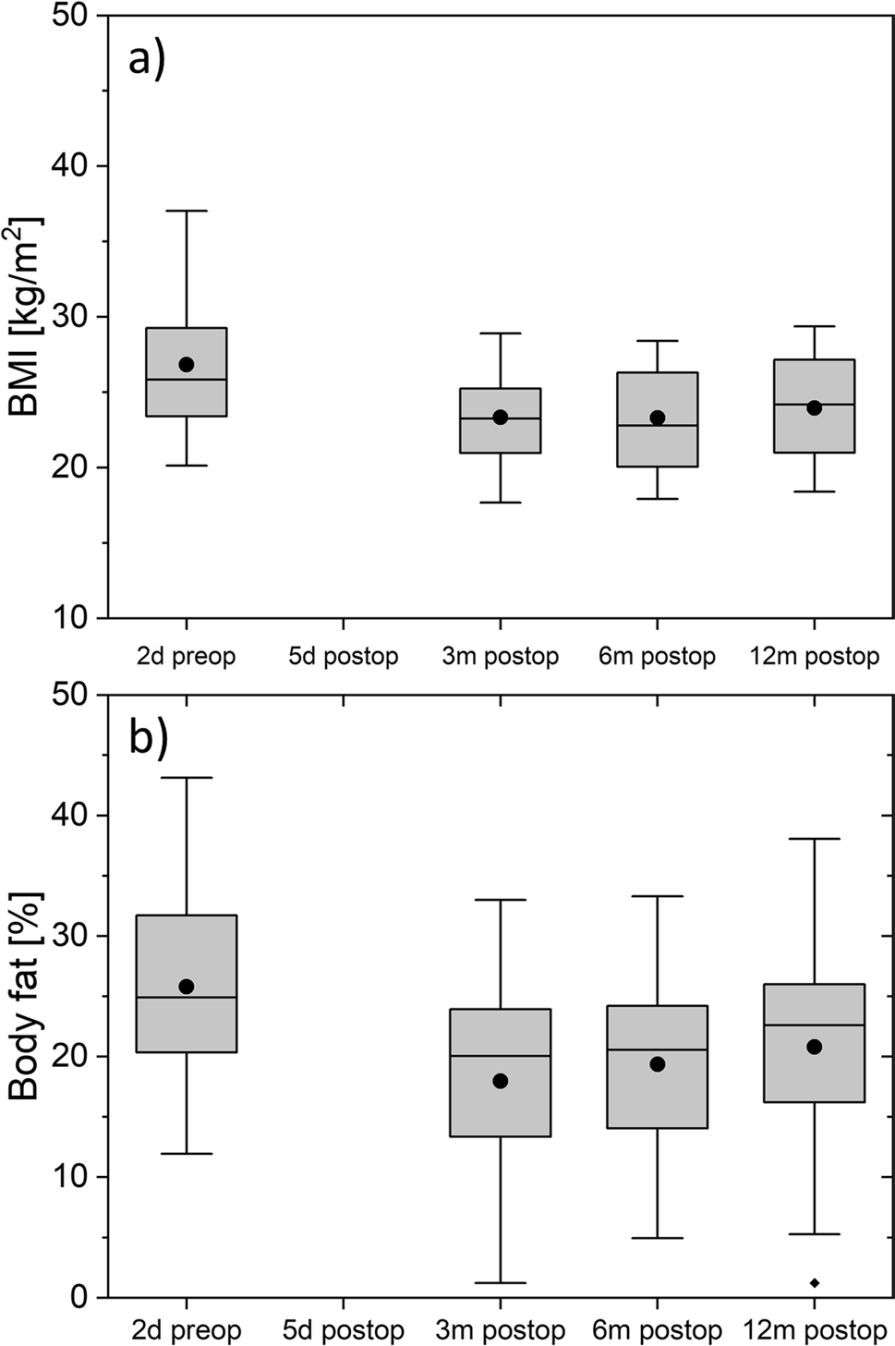
BMI (upper panel) and body fat (lower panel) for each follow-up. The boxes contain the 25% and the 75% quartile as well as the median, black dots depict the mean, and whiskers range from the 5% percentile to the 95% percentile.

**Figure 3:**
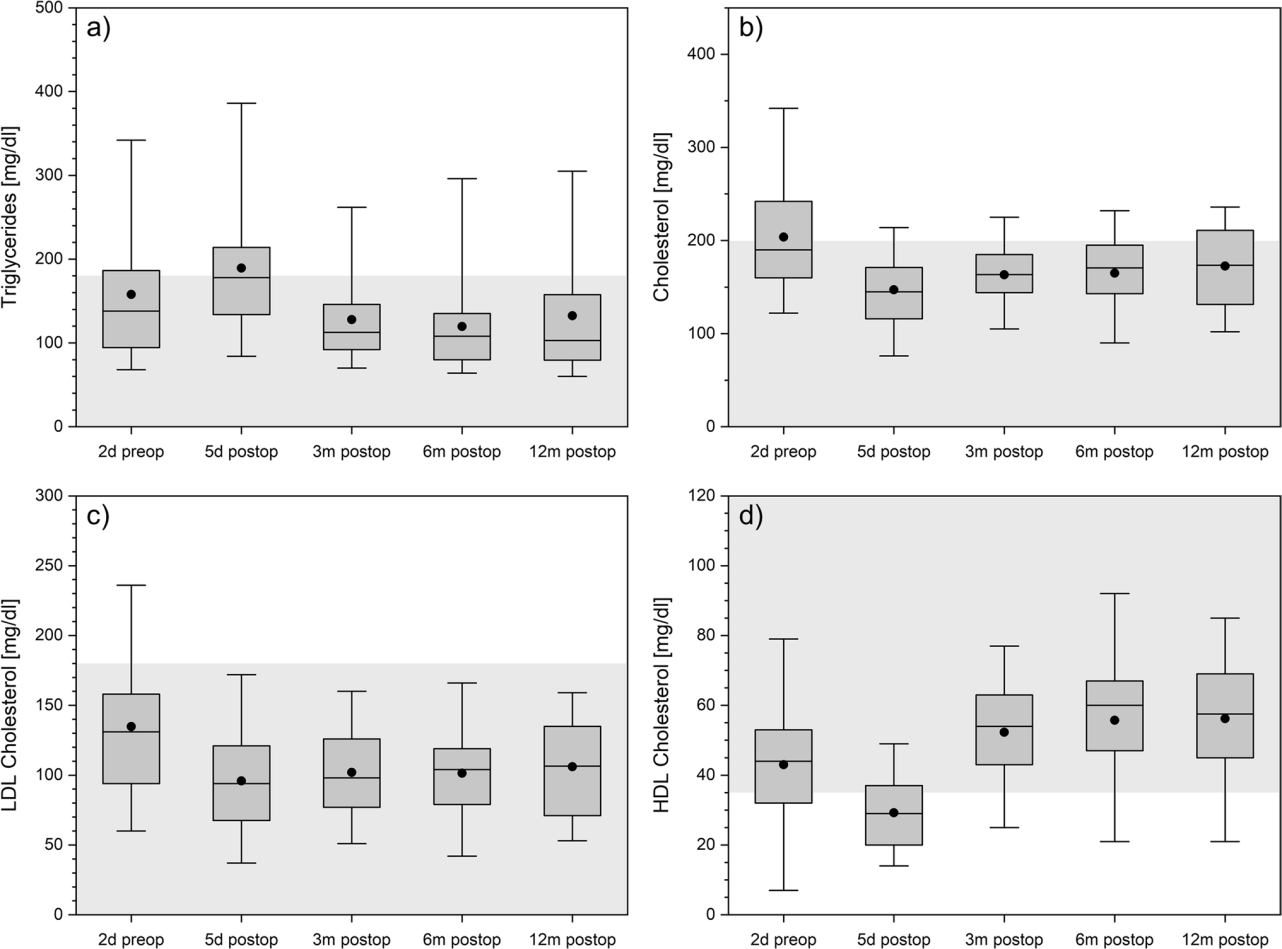
Triglycerides (panel **a**), cholesterol (panel **b**), LDL cholesterol (panel **c**), and HDL cholesterol (panel **d**) over time. The boxes contain the 25% and the 75% quartile as well as the median, black dots depict the mean, and whiskers range from the 5% percentile to the 95% percentile. Gray areas depict the normal range for each variable.

Due to routinely postoperative care in the intensive care unit the weight and body fat examinations were not carried out one week postoperatively, as relevant changes would not be expected anyway. A slight postoperative weight loss was detected over the 12 months of follow-up. A decrease of body fat proportion was detected over time which correlated with decreased BMI (Fig. 2a), a decrease in body fat as measured with the seven-site skinfold method (Fig. 2b) and lower cholesterol and LDL levels. Triglyceride, LDL and cholesterol were in normal range during the whole follow-up (Fig. 3a–c). HDL fell below normal values in early postoperative phase and already normalized at the 3-month follow-up (Fig. 3d).

### Albumin

**Figure 4:**
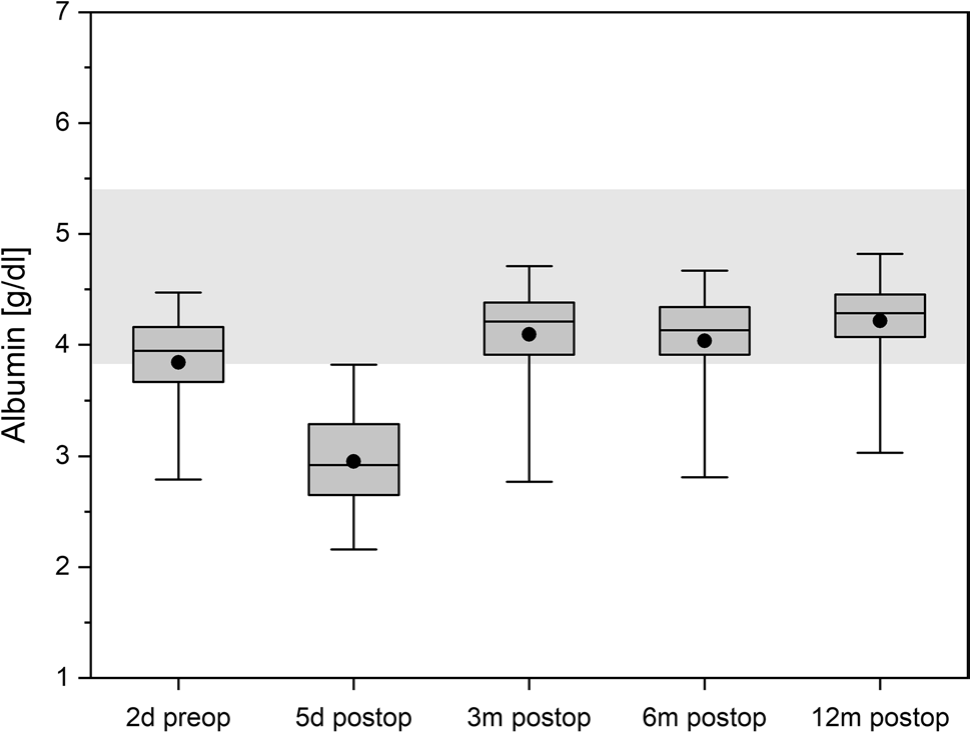
Albumin over time. The boxes contain the 25% and the 75% quartile as well as the median, black dots depict the mean, and whiskers range from the 5% percentile to the 95% percentile. Gray areas depict the normal range for each variable.

In the immediate postoperative phase, a significant drop could be detected which normalized at 3 months postoperatively and remained in normal range during the rest of the course (Fig. 4).

### Iron metabolism

**Figure 5:**
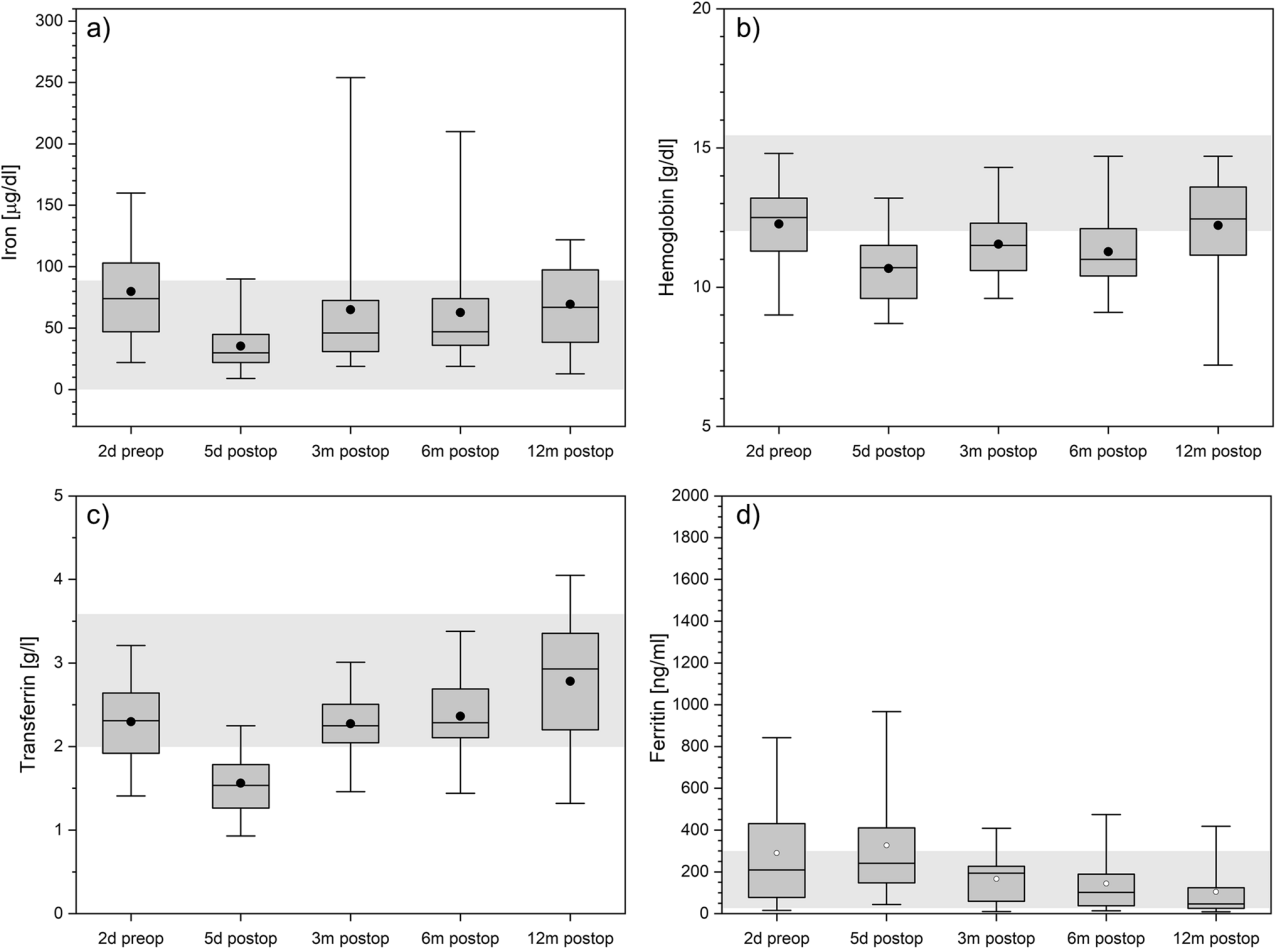
Iron (panel **a**), hemoglobin (panel **b**), transferrin (panel **c**), and ferritin (panel **d**) over time. The boxes contain the 25% and the 75% quartile as well as the median, black dots depict the mean, and whiskers range from the 5% percentile to the 95% percentile. Gray areas depict the normal range for each variable.

A decrease of iron, hemoglobin, and transferrin (Fig. 5a–c) and an increase of ferritin (Fig. 5d) one week postoperatively are noticeable. In the following laboratory examination, a steady decrease of ferritin to low normal values are found which correlated with low hemoglobin although hemoglobin levels after 12 months were close to preoperative baseline.

### Trace elements

**Figure 6:**
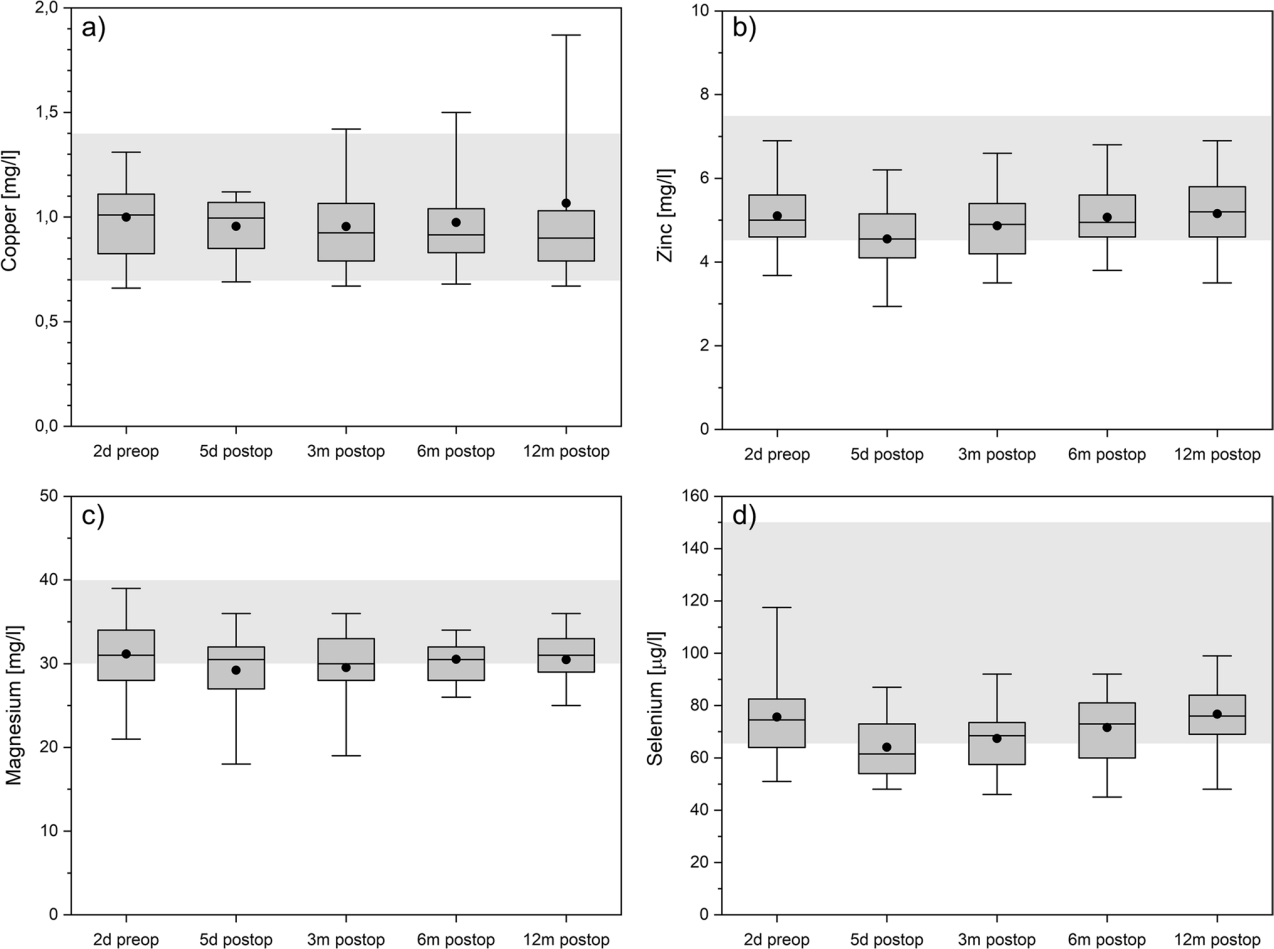
Copper (panel **a**), zinc (panel **b**), magnesium (panel **c**), and selenium (panel **d**) over time. The boxes contain the 25% and the 75% quartile as well as the median, black dots depict the mean, and whiskers range from the 5% percentile to the 95% percentile. Gray areas depict the normal range for each variable.

No significant changes in copper and zinc levels could be detected during the investigation period and values were well within normal range (Fig. 6a,b).

Magnesium levels were mostly in low normal ranges (Fig. 6c), with magnesium levels being lowest in the immediate postoperative phase. The same holds true for selenium (Fig. 6d).

### Bone metabolism

**Figure 7:**
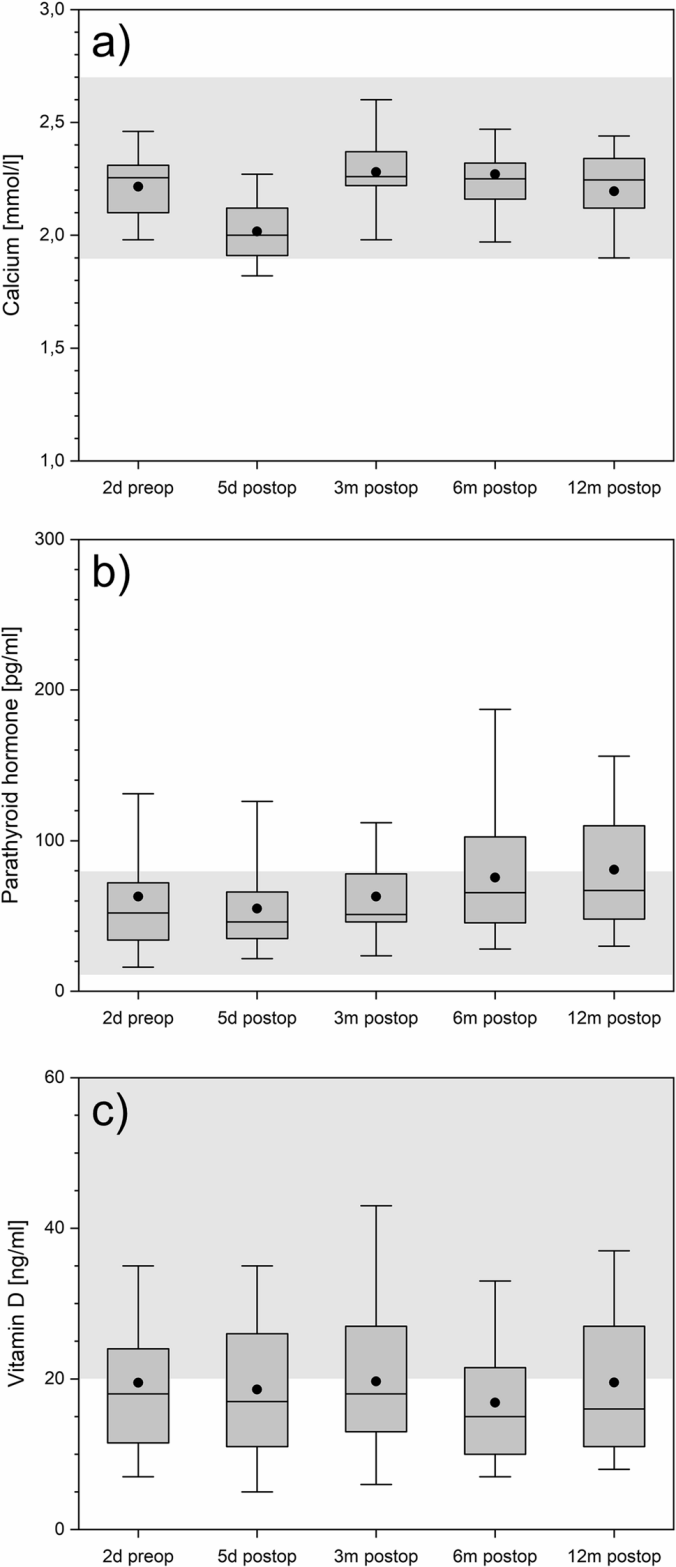
Calcium (panel **a**), parathyroid hormone (panel **b**), and vitamin D (panel **c**) over time. The boxes contain the 25% and the 75% quartile as well as the median, black dots depict the mean, and whiskers range from the 5% percentile to the 95% percentile. Gray areas depict the normal range for each variable.

Serum calcium is in most cases in normal range (Fig. 7a). This same effect can be shown for parathyroid hormone with values rising above the normal range especially from the sixth postoperative month (Fig. 7b). More than 50% of all patients suffer from severe vitamin D deficiency with levels in very low normal range or less than 20 ng/ml (Fig. 7c).

### Vitamin A

**Figure 8:**
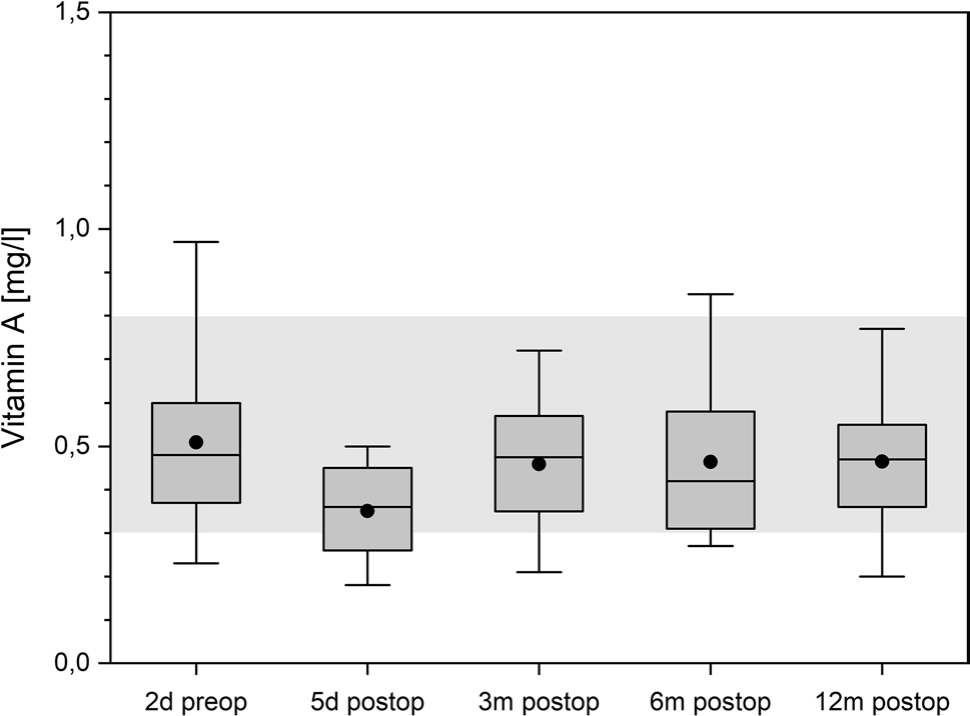
Vitamin A over time. The boxes contain the 25% and the 75% quartile as well as the median, black dots depict the mean, and whiskers range from the 5% percentile to the 95% percentile. Gray areas depict the normal range for each variable.

Preoperatively, Vitamin A status is in normal range and the lowest value is reached one week postoperatively (Fig. 8). In the following course its status is in normal range but in the IG group its serum concentration is at each time point higher than the CG although the preoperative baseline is higher in CG.

### Vitamin B1, B6, B12, homocysteine and folic acid

**Figure 9:**
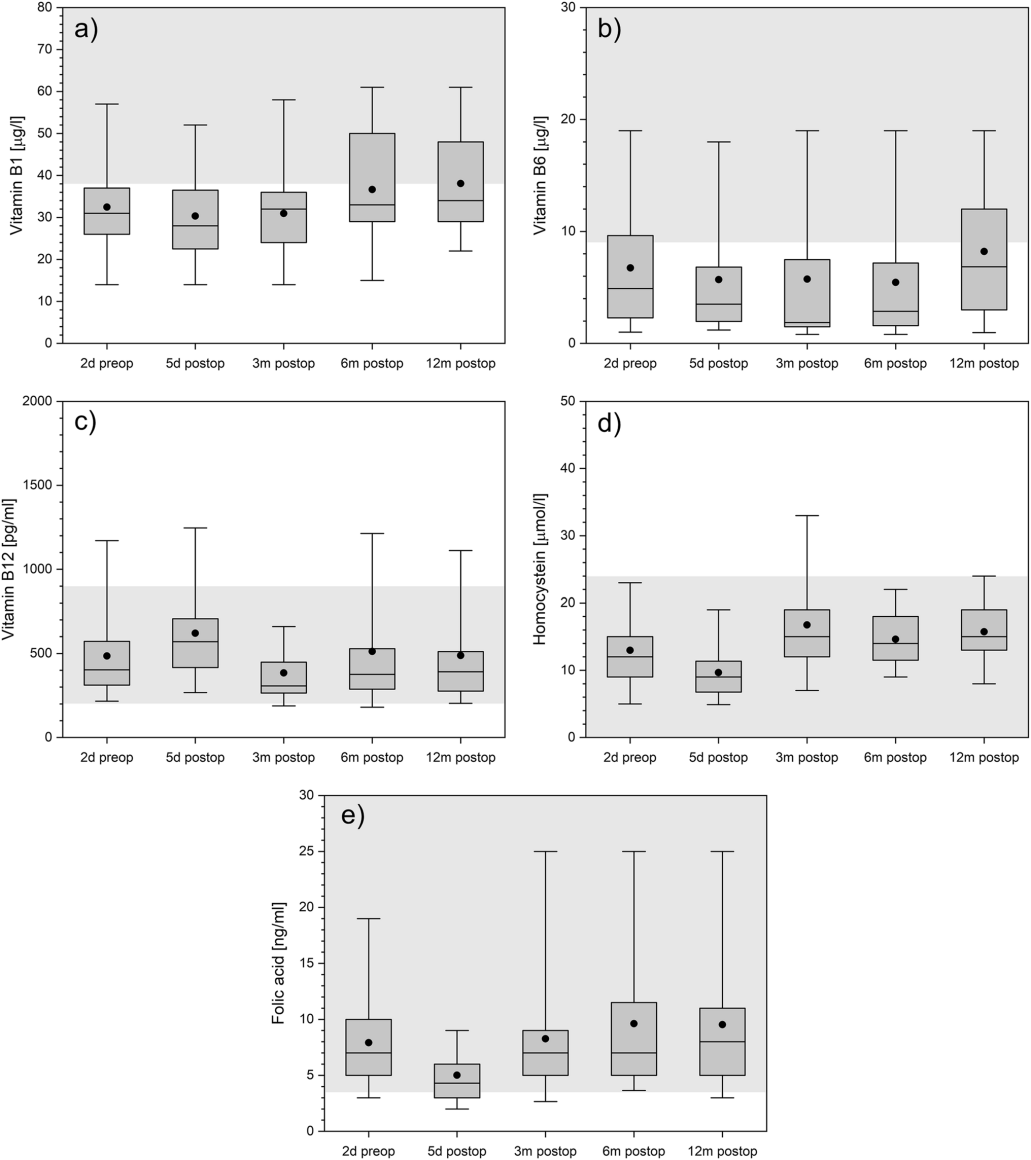
Vitamins B1 (panel **a**), B6 (panel **b**), and B12 (panel **c**), as well as homocysteine (panel **d**) and folic acid (panel **e**) over time. The boxes contain the 25% and the 75% quartile as well as the median, black dots depict the mean, and whiskers range from the 5% percentile to the 95% percentile. Gray areas depict the normal range for each variable.

At each time point the median vitamin B1 and B6 serum concentrations are lower than the bottom reference value indicating a vitamin B1 and B6 deficiency in general (Fig. 9a,b). However, a slight tendency towards normal range values can be seen for both in the last follow-up. Vitamin B12 stays within normal range throughout the whole study period (Fig. 9c). Homocysteine and folic acid levels are well within the normal range during follow-up period (Fig. 9d,e). However, there is a decrease in the early postoperative phase, and during the follow-up values are at even higher levels than preoperatively.

### Analysis with regards to the influence of physiotherapy

**Figure 10:**
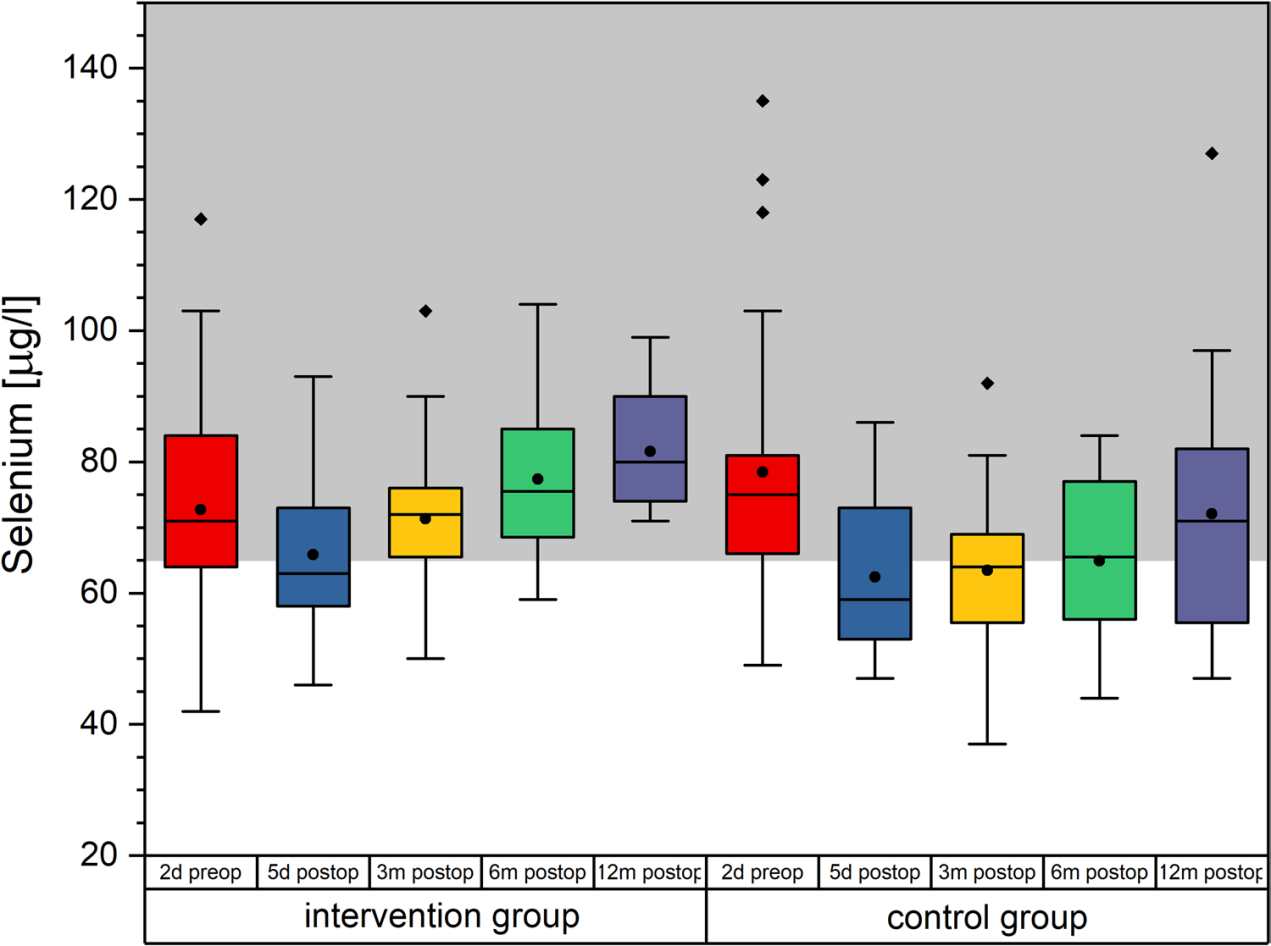
Selenium separately for the intervention and the control group over time. The boxes contain the 25% and the 75% quartile as well as the median, black dots depict the mean, and whiskers range from the 5% percentile to the 95% percentile. Gray areas depict the normal range for selenium.

When analyzed by physiotherapy cohort, significant differences between the IG and CG group for the parameters Cholesterol, HDL cholesterol and selenium at the 3, 6 and 12 months follow up, with all *p*-values ≤ 0.033, and effect sizes > 0.7 could be demonstrated (see also Table 2). However, it should be noted here that only for selenium the median for the IG patients was within the normal range at each time point, whereas median for the CG patients had levels below normal value during most of the postoperative phase (see Fig. 10). For all other parameters mentioned above, despite the differences between groups, both groups were either within the normal range or both were outside the normal range.

**Table 2:** Mean and 95% confidence interval for the three laboratory parameters showing significant differences in t-tests with regards to the influence of physiotherapy on the micronutritional status.

	Cholesterol (< 200 mg/dl)		HDL Cholesterol (> 35 mg/dl)		Selenium (65–150 µg/l)	
	IG	CG	IG	CG	IG	CG
2d preop	209.7 ± 25.9	197.3 ± 17.0	43.0 ± 7.8	43.1 ± 5.7	72.7 ± 6.0	78.4 ± 7.0
5d postop	155.5 ± 16.2	138.2 ± 16.8	29.6 ± 3.8	28.9 ± 4.1	65.9 ± 5.4	62.4 ± 4.9
3 m postop	***175.3* ± *11.5***	***149.7* ± *16.7***	***57.0* ± *5.8***	***47.3* ± *6.4***	***71.3* ± *6.6***	***63.4* ± *6.4***
6 m postop	***178.4* ± *15.4***	***149.6* ± *19.6***	57.8 ± 8.6	53.3 ± 9.3	***77.4* ± *6.0***	***64.9* ± *7.1***
12 m postop	***186.1* ± *19.3***	***158.9* ± *23.3***	***64.1* ± *10.5***	***48.3* ± *7.7***	***81.6* ± *4.5***	***72.1* ± *10.1***

Significant differences are marked by bold italics. IG: intensive group, CG: control group. Norm values are given in the brackets below the parameter name.

## Discussion

The analyses presented here of part of the data collected in our RCT mainly set out to examine the longitudinal changes in nutritional status after pancreatic surgery in a typical PD patient collective receiving no nutritional supplements.

It is well known that in the early postoperative phase, inflammatory processes and post-aggression metabolism take place including changes of acute phase proteins, which act as inflammatory markers. Some of the micronutrients assessed here play a crucial role in this process, including the so called positive acute phase reactants CRP, ferritin, and copper, as well as the negative acute phase reactants iron, transferrin, HDL, albumin, zinc, and selenium. The interaction of these reactants is essential for a physiological immune response^12^, as also confirmed by our data. Therefore, for evaluation of micronutritional status after surgery early postoperative changes of some micronutritional markers should be interpreted carefully. For valid assessments of long term changes, a period of four weeks should pass to ensure normalization of inflammatory processes.

In this light, the changes of iron metabolism can be well explained, especially one week postoperatively, and these results correspond well to findings in other studies^21^. In this regard, long term data after PD are spars and partly inconsistent, as ferritin or hemoglobin concentrations are often missing^11^. Since vitamin B12 and folic acid are in normal range during the complete observation period, it can be assumed that most patients suffer from iron deficiency anemia. It has been suggested that long term survivors could benefit from an iron substitution in case of fatigue and anxiety symptoms^9^. This is supported by our data.

Like iron, zinc and selenium are also well established as negative acute phase reactants, which explains the postoperative drop after one week. All these three trace elements are mainly absorbed in the duodenum and upper jejunum which are removed during PD. Therefore, it could be assumed that the surgical resection of these organs will lead to a postoperative deficiency. Zinc deficiency was reported in up to 68% of pancreas resected patients^22^ but there are also studies which do not report a zinc deficiency in their cohort^9^. Nevertheless, it seems that other compensation mechanisms play a role in the hemostasis of these elements, as serum concentration of zinc and selenium are in normal range in our patients during the later follow up. It has been assumed that, as anti-oxidative agents, selenium and zinc deficiency could contribute to chronic pancreatitis or anastomotic strictures and should be substituted after PD^23,24^. However, general substitution cannot be recommended by our data.

Magnesium is an essential mineral that is involved in the energy metabolism and the maintenance of muscle function. It is mostly resorbed in the upper gut. Surgical resections of the duodenum and proximal jejunum can lead to magnesium deficiency and even exocrine pancreas insufficiency can deteriorate magnesium levels by steatorrhea^11^. Additionally, adjuvant chemotherapy can increase hypomagnesemia^25^. However, near to 50% of all patents showed lower than normal magnesium levels. One reason for this might be the fact that we only analyzed extracellular nutrient levels, and at least for magnesium, this might influence the outcome. But clinically relevant hypomagnesemia after PD could not be observed in our collective. Therefore, no particular action seems to be warranted with regards to supplementation.

Resections of the pancreas can lead to a deficiency of fat-soluble vitamins such as vitamin A and D due to impaired fat digestion especially in cases of inappropriately substituted exocrine pancreas insufficiency^5,9,26,27^. Vitamin A concentrations were, except for a decrease shortly after surgery, mostly within normal range. The immediate postoperative drop is most probably due to immunological changes, as vitamin A is supposed to act as a negative phase reactant^28^.Therefore, supplementation of Vitamin A does not seem necessary, based on our collective. Intensified physiotherapy seems to positively influence its serum levels.

In contrast, we could see that most auf our patients were vitamin D deficient, even preoperatively. Vitamin D deficiency is very common in Germany and 57.3% of people aged 18–79 years have 25(OH)D serum levels below 50 nmol/L (20 ng/mL)^29^. Therefore, based on our data vitamin D deficiency cannot be associated to pancreas cancer per se, although, it has been postulated that vitamin D pathway gene variants may be associated with pancreas cancer^30^. However, irrespective of the intensity of physiotherapy, our patient collective suffered from vitamin D deficiency during the entire follow-up period, indicating that despite adequate pancreas enzyme substitution the vitamin D metabolism is impaired postoperatively.

Due to the largely normal vitamin A level, which is also a fat-soluble vitamin, it seems that not only maldigestion and malabsorption explain vitamin D deficiency but other factors such as low exposure to sunshine and low dietary vitamin D uptake and even chemotherapy might negatively influence its levels. In addition, it has been shown that pancreas resected patients need higher amount of vitamin D supplementation to reach serum 25(OH)D concentrations in range between 30 and 50 ng/ml^5^. Therefore, patients after pancreas resection should be monitored and adequately substituted with vitamin D to avoid bone demineralization, especially in cases where a longer survival time can be expected.

Since vitamin D levels show the lowest value after 6 months when patients undergo chemotherapy and increase again after 12 months, chemotherapy with gemcitabine seems to negatively affect the vitamin D status. This is probably even exacerbated by the fact that many patients also engage in less outdoor activity due to their poorer general condition during that period. In the case of normal calcium values at each time point and increasing vitamin D deficiency during the follow-ups, secondary hyperparathyroidism is acting as counter mechanism. Abundant data indicate an antiproliferative, apoptotic and antiangiogenic effect of vitamin D in different cancer types such as colon and breast cancer. But its general supplementation as adjuvant drug in pancreas cancer therapy is controversial as vitamin D supplementation can lead to hypercalcemia associated side effects^31^. In how far synthetic vitamin D analogs with fewer hypercalcemic and hypercalciuric side effects can be used as novel therapies in pancreatic cancer remains to be seen.

One of the advantages of the pylorus preserving Whipple operation is the preservation of the gastric antrum, and therefore, the prevention of subsequent loss of the intrinsic factor^32^, which is necessary for vitamin B12 absorption. Since our patients were exclusively operated using the pylorus preserving Whipple procedure, it is not surprising that no vitamin B12 deficiency could be detected. It should be mentioned, however, that a randomized comparison of vitamin B12 concentrations in patients with and without pylorus preserving Whipple operation is missing in the literature. Since the concentration of vitamin B12 is not subject to an inflammatory response, its concentration can also provide information about a deficiency immediately after surgery^12^ especially for differentiation between macrocytic and iron deficiency anemia.

Vitamin B6 or Pyridoxal-5´-Phosphatase is involved in various cellular processes including immune function, red cell metabolism or regulation of the nervous system. Additionally, cancer patients often manifest decreased levels of vitamin B6, and it seems that vitamin B6 deficient cancer cells appear to be much more resistant to stress-induced death, e.g., in lung cancer^33^, thus reducing the effects of chemotherapy. A recent meta-analysis suggested that vitamin B6 intake could significantly decrease pancreatic carcinoma risk^34^. Since our patients were consistently affected by vitamin B6 deficiency during the entire course, and in regard to the mentioned negative effects of its deficiency, patients should pay attention to foods with sufficient vitamin B6 concentration. In this context, nutritional counseling certainly plays an important role as well. How far its substitution could influence postoperative course and symptoms, such as anemia or fatigue syndromes, remains unclear.

Homocysteine is an intermediate product in the metabolism of the essential amino acid methionine, and hyperhomocysteinemia seems to be an independent risk factor for cardiovascular diseases. Folic acid, vitamin B12 and B6 deficiency inhibit the degradation of homocysteine, thus leading to increased levels of homocysteine^35^. It has been shown that chronic pancreatitis is associated with hyperhomocysteinemia and that low folate levels play a key role in the derangements of transmethylation and transsulfuration pathways involved in homocysteine metabolism^36^. The fact that folate and homocysteine levels were in reference range in the follow-up of our patients, suggests that pancreas resected patients are not significantly affected by metabolic changes of these elements.

Weight loss is very common among patients with pancreatic cancer, both at the time of diagnosis, as well as during the course of the disease. It is assumed that the depletion of body fat is one of the reasons for weight loss^37^. Decreased lean body mass and BMI correlate with higher perioperative morbidity, shorter progression-free and overall survival^38^. In our collective, 50% of patients had a preoperative BMI value lower than 30 kg/m^2^, and BMI and body fat steadily decreased during the observation period although patients reported normal food intake throughout the follow-up period. This finding confirms the known fact that pancreatic cancer and pancreas resected patients lose weight despite of “normal” nutritional habits and pancreas enzyme substitution. As concluded in other studies^39,40^, and based on our data, we also recommend that pancreas resected patients should be routinely supervised by a dietician, and they should be motivated to be physically active to prevent loss of muscle mass, to reduce negative impacts of weight loss on the course of the disease and on patient´s quality of life.

The comparison between IG and CG patients was performed as a supporting analysis to evaluate if malnutrition induced fatigue might have hindered patients in both groups in working out if they had wanted to. Effect sizes indicate, that sample size was adequate, although sample size calculation was based on the primary endpoint of the study. The only significant differences between both groups with regards to the examined micronutrients in this study was in selenium, so that ultimately, we can state that patients in both groups showed a comparable nutritional status in our sample. Therefore, we can conclude, that all patients should have been equally able to perform their respective physiotherapy regimen.

The obvious limitation of this study was the relatively small sample size, as the study sample size was calculated with the primary endpoint of the RCT (QoL with respect to intensive or standard physiotherapy) in mind. Therefore, no meaningful statistical evaluation could be conducted for each follow-up time point, to investigate if parameters differed significantly from normal range. However, our descriptive approach provides a valid basis for the recommendations made here. In addition, for the statistical tests conducted to evaluate possible differences between both physiotherapy groups, effect sizes indicate that in those cases the sample size was adequate. Second, a detailed postoperative evaluation of nutritional intake or a diet protocol as implemented in other studies^41^, was not performed. However, the patients were urged to eat a normal diet based on the above-mentioned ESPEN guidelines^15^, and the usual monthly phone calls and visits included open questions with regards to appetite and food intake. This allowed us to evaluate micronutritional status based on the usual dietary behavior of the patients, and therefore our data represent the status quo of the nutritional status of a typical patient collective after PD. Indeed, our data are thus unbiased by changes in food intake, which might result from respective monitoring.

The strength of this study lies in the very homogenous sample (only patients with pylorus preserving pancreatic resection were included), the rigorous sampling that comes along with conducting a RCT, and the long-term follow-up of 12 months. In addition, an insufficient supply of fat-soluble nutrients could be excluded due to the adequate supplementation with pancreatic enzymes. These points add to generalizability of the study results, such that the data reflect the clinical situation after pancreatic resection very accurately.

## Conclusions

To the best of our knowledge, this is the first study evaluating the micronutrient status of pancreas resected patients after standardized operation technique with a follow-up of 12 months. Taken together, we could demonstrate a deficiency of vitamin D, B1, B6, iron and iron associated anemia in a typical patient collective which is not specifically monitored with regards to food intake. Surgeons should keep in mind that most of the micronutrients are resorbed in the upper jejunum and extended resections should therefore be avoided. Vitamin D and iron should be supplemented postoperatively in the long term, and vitamin B1 and B6 substitution should be considered in symptomatic patients. Since all other laboratory parameters evaluated here are subject to large interindividual differences, a professional dietician care is recommended to ensure adequate care of pancreas resected patients. For future micronutrition studies it should be kept in mind that most of the micronutrients react as (negative) acute phase elements. Therefore, reliable assessment of their serum concentration is only possible after acute inflammatory processes have normalized and are thus not meaningful in the first few postoperative weeks. Effects of micronutritional status on other patient related parameters such as quality of life still have to be evaluated.

## Supplementary Information

Supplementary Information.

## Data Availability

The data contains sensible patient data, which albeit being anonymized might be used to identify certain patients with rare combinations of symptoms. Therefore, data is only available upon reasonable request to the corresponding author.
