# Supplementary material for: Micronutritional status after pylorus preserving duodenopancreatectomy: analysis of data from a randomized controlled trial
Source: Sci Rep. 2021 Sep 16;11:18475. doi: 10.1038/s41598-021-97438-6 (PMC8445937; doi:10.1038/s41598-021-97438-6)
Supplement: Supplementary file 1 — Supplementary Information. [file 41598_2021_97438_MOESM1_ESM.docx]

**Supplemental Table 1: Description of the test kits used for the laboratory parameters**

| **Parameter** | **Manufacturer** | **Name of the measurement platform** | **Normal range** | **Range of the intra assay coefficient of variation as a function of the mean value** | **Range of the inter assay measurement error with the RMS method as a function of the mean value*** |
| --- | --- | --- | --- | --- | --- |
| Albumin | Siemens | Advia Chemistry | 3.8-5.4 g/dl | 1.0-1.6 | 0.09-4.5 |
| Calcium | Siemens | Advia Chemistry | 1.9-2.7 mmol/l | 0.6-0.9 | 1.2-5.0 |
| Cholesterol | Siemens | Advia Chemistry | < 200 mg/dl | ~0.3 | 0.7-6.0 |
| Iron | Siemens | Advia Chemistry | 35-140 µg/dl | 0.5-0.9 | 0.5-3.9 |
| Ferritin | Siemens | Advia Chemistry | 20-300 ng/ml | 2.1-3.0 | 3.5-10.0 |
| Folic acid | Siemens | Advia Centaur | > 3.5 ng/ml | 4.5-7.9 | 6.2-37.7 |
| HDL cholesterol | Siemens | Advia Chemistry | < 180 mg/dl | 0.7-2.3 | 0.9-15.4 |
| Homocysteine | Siemens | Advia Centaur | < 24 µmol/l | 2.3-4.4 | 6.3-15.5 |
| Hemoglobin |  |  | 12-15.4 g/dl |  |  |
| LDL cholesterol | Siemens | Advia Chemistry | > 35 mg/dl | 0.6-0.8 | 1.9-15.6 |
| Magnesium | Immundiagnostik | -- | 30-40 mg/l | ~3.7 | -- |
| Parathyroid hormone | Siemens | Advia Chemistry | 10-80 pg/ml | 3.4-5.2 | 1.2-27.9 |
| Selenium | Immundiagnostik | -- | 65-150 µg/l | ~6.7 | -- |
| Transferrin | Siemens | Advia Chemistry | 2-3.6 g/l | ~1.1 | 1.5-8.1 |
| Triglyceride | Siemens | Advia Chemistry | < 180 mg/dl | 0.3-0.9 | 0.7-6.0 |
| Vitamin B12 | Siemens | Advia Centaur | 200-900 pg/ml | 2.4-5.0 | 5.0-15.8 |
| Vitamin A | Immundiagnostik | -- | 0.3-0.8 mg/l | ~1.5 | -- |
| Vitamin B1 | Immundiagnostik | -- | > 38 µg/l | ~7.2 | -- |
| Vitamin B6 | Immundiagnostik | -- | > 9 µg/l | ~15.8 | -- |
| Zinc | Immundiagnostik | -- | 4.5-7.5 mg/l | ~5.2 | -- |
| 25-OH Vitamin D | IDS | iSYS | > 20 ng/ml | 3.8-5.0 | 4.6-35.8 |

* For the inter assay values the ranges of the root mean square (RMS) errors of the measurement are given if available. RMS errors were calculated for quality assurance reasons at various instances throughout the study period. Mean values for the RMS errors have all been within the tolerance range of less than 10%.
